# Burnout Syndrome and Work-Related Stress in Physical and Occupational Therapists Working in Different Types of Hospitals: Which Group Is the Most Vulnerable?

**DOI:** 10.3390/ijerph17145001

**Published:** 2020-07-11

**Authors:** Ju-Hyun Kim, Ae-Ryoung Kim, Myung-Gwan Kim, Chul-Hyun Kim, Ki-Hoon Lee, Donghwi Park, Jong-Moon Hwang

**Affiliations:** 1 Department of Rehabilitation Medicine, Kyungpook National University Hospital, Daegu 41944, Korea; kjoohyun88@gmail.com (J.-H.K.); ryoung20@hanmail.net (A.-R.K.); chgim@knu.ac.kr (C.-H.K.); 2 Department of Rehabilitation Medicine, School of Medicine, Kyungpook National University, Daegu 41944, Korea; 3 Graduate School of Public Health, Kyungpook National University, Daegu 41566, Korea; curein@naver.com; 4 Mompyeonhan Rehabilitation Clinic, Daegu 42401, Korea; qu291@naver.com; 5 Department of Physical Medicine and Rehabilitation, Ulsan University Hospital, University of Ulsan College of Medicine, Dong-gu, Ulsan 44033, Korea

**Keywords:** burnout syndrome, vulnerable group, physical therapist, occupational therapist, gender, hospital size, age

## Abstract

Because of the nature of their work, physical and occupational therapists are at high risk of burnout, which is associated with decreased job satisfaction, medical errors, and mental wellbeing in healthcare professionals. To well manage and minimize potential impact of burnout, risk factors should be determined. This study examined burnout and job stress in physical and occupational therapists in various Korean hospital settings. Physical and occupational therapists from several rehabilitation facilities in South Korea completed a survey between March–May 2019. A set of questionnaires, including the Maslach Burnout Inventory and Job Content Questionnaire, were distributed to all participants. In total, 325 professionals (131 men and 194 women) were recruited. Burnout and work-related stress differed significantly according to several factors. Hospital size, gender, and age were the main contributory factors affecting at least two dimensions of the questionnaires. The more vulnerable group consisted of female therapists in their 20s at small- or medium-sized hospitals with low scores for quality of life. High levels of job stress and burnout were observed in female therapists in their 20s at small- or medium-sized hospitals. Hospitals and society should create suitable environments and understand the nature of therapists’ work to improve healthcare.

## 1. Introduction

Burnout is a state of physical and emotional exhaustion involving the development of both a negative self-concept and a negative attitude toward one’s job. This concept was first described in 1975 by Herbert Freudenberger, an American psychologist, who defined it as follows: “Burnout” is “to fail, wear out, or become exhausted by making excessive demands on energy, strength, or resources” [1].

Maslach and Jackson later described burnout syndrome in three major dimensions: emotional exhaustion (EE), depersonalization (DP), and lower perception of personal achievement (PA) [2]. Emotional exhaustion refers to feelings of emotional overextension and being drained by others. Depersonalization refers to a callous response towards the people receiving the service. Reduced personal accomplishment refers to a decline in one’s feelings of competence and a reduction in successful achievement in one’s work with people. Recently, job burnout is commonly referred to as a negative psychological reaction caused by an increase in chronic work-related stress [3]. In general, many factors may significantly contribute to burnout, including reward, community, fairness, values, and job–person incongruity [2,4].

Healthcare professionals have been described as particularly vulnerable to burnout [1,5]. Physical and occupational therapists, among other healthcare personnel, are at high risk of burnout syndrome because of the nature of their work. Therapists are in daily contact with the physical and psychological pain of clients as they face various states of disability. This naturally triggers emotional responses and may lead therapists to defend themselves by distancing themselves from relationships with their patients [6]. As they spend the majority of the workday deeply involved with their patients, the therapy they provide is emotionally, physically, and intellectually challenging [1,6,7,8,9,10].

In healthcare professionals, burnout has been associated with a lack of concentration [11], drug and alcohol abuse, increased depression, and suicide [12], leading to poor quality of life [13]. In addition, burnout can lead to high turnover resulting from decreased job satisfaction, which burdens other team members [14,15,16,17]. Moreover, medical errors increase with burnout, which hinders the quality of medical treatment [17,18]. Further, the loss of medical personnel due to burnout can lead to poor medical quality and socioeconomic loss [19,20,21].

Various studies have been conducted to examine burnout syndrome in physical and occupational therapists [18,22,23,24]. A study conducted by Corrado and colleagues confirmed that physical therapists are at a high risk of developing burnout syndrome. However, the total number of subjects was 118, and the sample size was relatively small [23]. An article by Deckard and colleagues examined role stress on physical therapists but also had flaws in sampling size [18]. And a few existing studies have explored burnout according to hospital characteristics. The working environment is expected to exert a significant effect on the frequency and degree of burnout [25], and a multicenter study that can confirm the results according to the difference in hospital character is essential. In addition, because of the recent social demand for rehabilitation, a growing number of hospitals employ both physical and occupational therapists. However, previous studies included either physical or occupational therapists [8,9,10,11,18,23].

To well manage and minimize the potential impact of burnout, it is important to understand which groups are particularly vulnerable to burnout. And it is essential to investigate the risk factors in finding vulnerable groups. Therefore, the current study aims to investigate the level of burnout and job stress among physical and occupational therapists in Korea and identify which type of people are vulnerable to burnout. To find vulnerable groups, analysis of factors, such as hospital and personal characteristics, has been conducted.

The following research questions were posed:

(a) What is the level of burnout and job stress among physical and occupational therapists?
(b) Which groups are particularly vulnerable to burnout?
(c) Which demographic or hospital characteristics are related to burnout?

By identifying the vulnerable groups, it could help to manage the group well with caution, solve working environmental problems, and ultimately improve the overall quality of healthcare provision in South Korea.

## 2. Materials and Methods

### 2.1. Sample

Subjects were recruited from 4 hospitals with over 100 beds and 5 hospitals with under 100 beds in the Republic of Korea between March–May of 2019. The following inclusion criteria were adopted: therapists with a bachelor’s degree in physiotherapy or occupational therapy, and those in direct contact with patients being under professional activity.

After visiting the rehabilitation unit of each hospital, the therapist who met the inclusion criteria were asked for written consent to participate in the study, and a set of questionnaires was distributed. Participants were asked to complete a brief, anonymous questionnaire, which was followed by assessment. It contained items pertaining to general demographic characteristics, such as gender, age, annual income, and job group. In addition, it included items regarding job characteristics, such as hospital size, and personal health behaviors, such as smoking, drinking, exercise, and leisure time.

The job group was divided into two types: physical therapist and occupational therapist. The physical therapists perform therapeutic exercises, such as lower extremity strengthening, range of motion (ROM) exercise, gait training, trunk balance training, and manual therapy for musculoskeletal injury. Occupational therapists conduct treatment programs to perform activities of daily living (ADL) and perform upper extremity strength training, ROM exercise, dysphagia therapy, ADL training, and cognitive rehabilitation.

Regarding hospital size, hospitals with more than 100 beds are classified as ‘general hospitals’ under the Korean medial law, many medical departments are often gathered, and various employee welfare, including industrial accident insurance, is well guaranteed. On the other hand, in hospitals with less than 100 beds, which are classified as ‘hospitals’, the number of departments is usually 1 or 2, and employee welfare is often not as well guaranteed as that of large hospitals. Therefore, hospitals were classified as large if they had ≥100 beds and as small- or medium-sized if they had <100 beds. The duties of therapist were the same regardless of the size of the hospital.

Three hundred sixty-seven professionals who met the inclusion criteria were prepared, and 325 (131 men and 194 women) were analyzed, with the exception of 42 who refused to respond or the data were unable to be analyzed due to poor responses.

### 2.2. Maslach Burnout Inventory

The Maslach Burnout Inventory (MBI) designed by Maslach and Jackson was used to measure burnout [2,26] and consists of 22 items. Cronbach’s α was 0.76 when the questionnaire was designed and 0.87 in the current study. The scale has three subscales, each of which measures a dimension of burnout syndrome: Emotional Exhaustion (EE), Depersonalization (DP), and Personal Achievement (PA). EE measures emotional exhaustion experienced at work (9 items); DP measures the presence of negative attitudes and feelings toward the recipients of services (5 items), and PA measures the presence of feelings of low accomplishment and professional failure (8 items). Cronbach’s α for each subscale of EE, DP, and PA were 0.863, 0.688, and 0.811, respectively. All responses were provided using a Likert scale ranging from 1 (totally disagree)–5 (totally agree). The scores for each subscale were summed and divided by the number of items to obtain a mean score. Higher EE and DP scores indicate high levels of burnout, while higher PA scores reflect low levels of burnout.

### 2.3. Job Content Questionnaire

The Job Content Questionnaire (JCQ) [27,28], which contains 22 items based on the job strain model, was used to evaluate the risk of developing work-related stress. It includes dimensions regarding quantitative (1) psychological job demands (5 items); (2) decision latitude, including skill discretion (6 items) and decision authority (3 items); and (3) social support including coworker (4 items) and supervisor (4 items) support. Responses were provided using a 4-point Likert scale, ranging from 1 (strongly disagree)–4 (strongly agree). The item scores were summed and divided by the total number of items to obtain a mean score. Higher scores indicate lower levels of job stress. In the current study, Cronbach’s α for the scale was 0.79. 

### 2.4. Short Form 36

The Short Form 36 (SF-36) was used to evaluate health-related quality of life (QOL) [29]. This scale has been shown to be a credible and reasonable instrument for measuring QOL [30]. Existing studies dealing with QOL in health care personnel used SF-36 [13,31,32], and it is considered valid, reliable, comprehensive, brief, and potentially useful for individual patient applications [33]. Therefore, SF-36 was also used in this study. The questionnaire contains 8 itemized categories and 36 questions. The 8 categories are as follows: (1) physical functioning (e.g., walking or lifting), (2) role function-physical (e.g., limitations in the ability to perform usual activities), (3) body pain (e.g., level of body pain or discomfort), (4) general health perceptions (e.g., global evaluation of health), (5) vitality (e.g., energy level or fatigue), (6) social functioning (e.g., impact of health or emotional problems on social activities), (7) role function-emotional (e.g., impact of emotional problems on work or daily activities), and (8) mental health (e.g., anxiety, depression, and a sense of psychological wellbeing). The raw data from the 8 categories were converted into scores, categorically combined, and converted to a scale ranging from 0–100 using the Rasch measurement model. Higher combined scores represent better QOL. The first 4 categories were grouped as the physical component summary, and the final 4 categories were grouped as the mental component summary.

### 2.5. Data Analysis

Data were analyzed using open-source software R version 3.6.1 (R Foundation, Vienna, Austria). To demonstrate distribution, frequency analysis was used for general and job characteristics. And independent *t*-tests and ANOVA were used to compare job stress and burnout according to various variables. After identifying vulnerable subgroups with severe burnout and high job stress (in the previous analysis), SF-36 score was compared between the vulnerable group and the entire sample through independent *t*-test. 

### 2.6. Ethical Considerations

The protocol used in the study was approved by the institutional review board at the hospital (IRB No. 2019-03-006-004). Participation was voluntary, and written consent was obtained from all participants. All information was considered confidential.

## 3. Results

### 3.1. Demographic Characteristics

Of the 325 participants, 194 (59.7%) were women, 178 (54.8%) were aged 20–29 years, 232 (71.4%) were single, and 227 (69.8%) worked at small hospitals. Regarding job type, 216 (66.5%) and 109 (33.5%) were physical and occupational therapists, respectively.

### 3.2. Burnout and Job Stress by Hospital Size

Participants’ burnout and job stress differed significantly according to hospital size (Table 1). The mean JCQ score was 1.88 ± 0.27 for therapists working at hospitals with ≥100 beds and 1.80 ± 0.26 for therapists working at hospitals <100 beds, indicating that therapists working at small- or medium-sized hospitals showed higher levels of job stress relative to those working in large hospitals.

The mean EE score was 1.74 ± 0.67 at large hospitals and 2.07 ± 0.65 at small- or medium-sized hospitals. DP scores in small- or medium-sized hospitals (1.38 ± 0.59) were higher relative to those in large hospitals (0.98 ± 0.58). PA scores in small- or medium-sized hospitals (3.08 ± 0.06) were lower relative to those in large hospitals (3.47 ± 0.51). In other words, therapists in small- or medium-sized hospitals showed higher burnout levels relative to those of therapists in large hospitals for all three MBI dimensions.

### 3.3. Groups Vulnerable to Burnout and Job Stress

To identify those vulnerable to burnout and job stress, changes in job stress according to various variables besides hospital size were examined, as shown in Table 2. In hospitals with ≥100 beds, job stress did not differ significantly according to these factors. However, in hospitals with <100 beds, job stress levels differed according to gender, annual salary, and religion, and stress levels were higher in women, those with lower annual income, and those without religion.

The MBI subscale data for various variables are shown in Table 3 and Table 4. In large hospitals, PA scores in women were higher relative to those in men. With respect to age, participants in their 20s showed higher EE, DP, and PA scores relative to those of participants aged >30 years. Regarding annual income, lower income was associated with higher EE and PA scores. Married therapists showed lower DP and PA scores relative to those of single therapists. In addition, temporary employees showed higher DP scores relative to regular workers, and those with no experience of leaving a job showed higher EE and PA scores relative to those with this experience. PA scores differed significantly according to educational level and religion. Therapists with <3 years of work experience showed the highest PA scores.

At hospitals with <100 beds, gender, age, salary, educational level, marital status, job type, type of employment, and years of experience affected burnout levels (Table 4). Women showed higher EE and DP scores relative to men. Regarding age, the EE score was higher, and PA score was lower in participants in their 20s relative to those participants aged ≥30 years, indicating severe burnout. Lower incomes were associated with higher EE, DP, and PA scores. Highly educated people tended to show higher PA scores relative to those with lower educational levels, and single people showed higher EE scores relative to married people. Physical therapists showed lower PA scores relative to occupational therapists, and temporary workers showed higher DP scores relative to regular workers. EE scores were highest in those with 3–6 years of work experience. 

In summary, therapists in small- or medium-sized hospitals tended to show higher job stress and burnout levels relative to those in large hospitals. In addition, women in their 20s were shown to be particularly vulnerable to job stress and burnout. Hospital size, gender, and age exerted effects on more than one domain and were representative to some extent, although many other factors also affect burnout.

**Figure 1:**
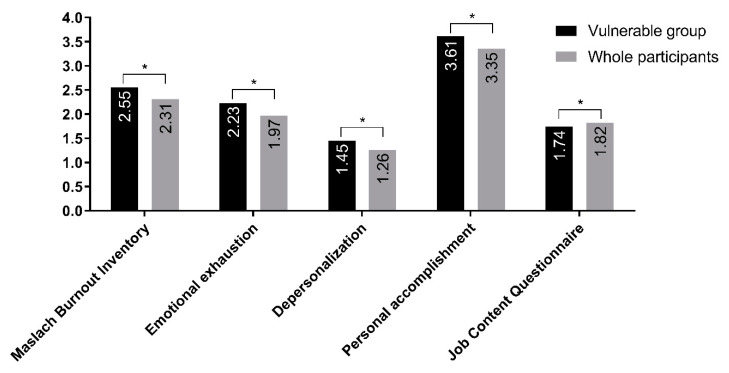
Comparison of Maslach Burnout Inventory (MBI) and Job Content Questionnaire (JCQ) scores between vulnerable group and overall participants. *—*p* < 0.05.

It is obvious that the vulnerable group has more job stress and burnout, but to quantitatively compare the specific degree, their scores were compared to those of the overall sample (Figure 1). Burnout for all three dimensions (EE, DP, and PA) and job stress was more severe in the vulnerable group relative to the overall sample.

**Figure 2:**
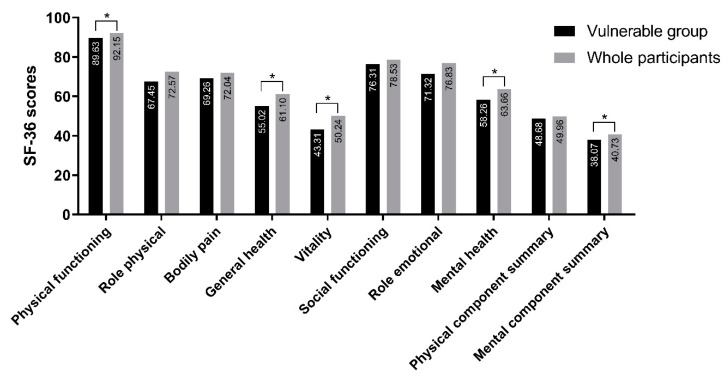
Comparison of Short Form 36 (SF-36) subscale and summary scores between vulnerable group and overall participants. *—*p* < 0.05.

It is clear that the vulnerable group also have lower QOL, but to determine the specific extent, SF-36 was compared to those of the overall sample. As shown in Figure 2, scores for the four SF-36 domains, physical functioning, general health perceptions, vitality, and mental health, were significantly lower in the vulnerable group relative to the overall sample. Physical component summary scores did not differ significantly between the vulnerable group and the overall sample, while mental component summary scores in the vulnerable group were lower relative to those in the overall sample. This result indicates that the vulnerable group experienced not only high job stress and burnout levels but also poor QOL.

## 4. Discussion

This study revealed that female therapists in their 20s working at small- or medium-sized hospitals are vulnerable to burnout and identified individual and work environment-related factors. The study by Lindqvist and colleagues (2015) reported that working in a large hospital induced greater stress [25], but the current study showed opposing results. The differences in job stress and burnout according to hospital size could have occurred because of differences in the hospitals’ working environments including the welfare system and patient groups encountered during work. Organizational factors also play a crucial role in the health professional’s burnout [34], and the difference in organizational culture according to the size of the hospital would also have an effect on the level of burnout. In addition, personal characteristics, such as pride at being part of a larger hospital, could have exerted effects. In this study, because each hospital had different characteristics (e.g., the proportions of men and women and ages of staff members), a multiple regression analysis was performed to calibrate the variables. Significant differences in job stress and DP and PA scores were observed, indicating that hospital size exerted independent effects on job stress and burnout.

Differences in job stress and burnout according to gender in large hospitals were not as prominent as those reported in previous studies [35,36,37], and women’s PA scores were higher relative to those in men. In contrast, in small- or medium-sized hospitals, women’s job stress levels and EE and DP scores were higher relative to those of men. This finding could have occurred because issues, such as gender discrimination, in the work environment varied according to hospital size. Reflecting the current social trend, discrimination between men and women in terms of salary and promotion opportunities has declined in large, but not in small- or medium-sized hospitals [38,39].

As for the age classification, because of Korea’s short history of physical/occupational therapy, many therapists are young. There were 178 therapists in their 20s, 121 in their 30s, 26 in their 40s, and 26 in their 40s or 50s. Statistical limitations have arisen in classifying people in their 40s and 50s as independent groups, so this study divided them into two groups: 178 people under 30 years old, and 147 people over 30 years old. Regarding age, participants in their 20s showed higher EE scores relative to older participants, regardless of hospital size. On the other hand, in small- or medium sized hospitals, PA scores observed in therapists in their 20s were lower, relative to those of older participants, and the opposite was observed in large hospitals. The result for the small- or medium-sized hospitals is consistent with the findings of a study conducted by Coward, in which older age was associated with higher levels of job satisfaction [40]. This could be related to the skills required in the work.

Concerning differences in job stress and burnout according to salary at large hospitals, the annual salary was investigated in Korean money, “won”, and 1 million won is currently worth between 800–900 US dollars. EE scores were highest in the group with an annual salary of <20 million won, but this finding was limited statistically because this group included only four participants. In the small- or medium-sized hospitals, EE and DP scores in the group with annual salaries of 20–30 million won were the highest, and PA scores in the group with incomes of >50 million won were the lowest. In general, lower pay was associated with greater risk of burnout. This finding is similar to those reported in a study conducted by Lee [41], in which higher salaries were associated with higher job satisfaction.

Temporary workers were found to have higher DP scores relative to regular workers, regardless of hospital size. This finding could have occurred because of stable working patterns. According to Seo [42], the fact that regular employees receive higher salaries relative to temporary workers, could also have led to this result.

Comparing the vulnerable group and the overall sample revealed significant differences not only in job stress and burnout but also in QOL, which corresponded to the results of a previous study on burnout and QOL [13].

This study found that female therapists in their twenties working in hospitals with less than 100 beds are most vulnerable to burnout. In this regard, further evaluation at small- or medium-sized hospitals is needed to ascertain whether members of the vulnerable group were being sexually discriminated against, had welfare problems, or had excessive workload. In particular, it is necessary to check whether discrimination in salary or promotion is present according to gender. By preventing gender discrimination, or supplementing insufficient welfare policies to vulnerable group, job satisfaction will be enhanced and further quality of medical care can be improved. In addition, the government should also provide financial/institutional support to help small- and medium-sized hospitals manage the burnout of therapists.

The current study was subject to a few limitations. First, although the measurement tools used in the study were standardized, data were collected according to the self-reporting method. Therefore, the risk of responder bias cannot be ruled out. Further, the study was cross-sectional and measured factors related to job stress and burnout simultaneously. Several factors were found to be relevant, but no causal relationships could be inferred. Further prospective research is required to identify the causal relationships between these factors and job stress, burnout, and QOL. Differences in the work environment, which is based on 100 beds, are caused by the Korean medical law, and since the standards vary by country, there is a limit to the same application in other countries. However, apart from the specific number of 100 beds, it would be more practical to think of it as a standard for large hospitals, or small and medium hospitals that fit each country’s situation. Lastly, in some analyses, the sampling size was too small, which was statistically limited. For example, in hospitals with more than 100 beds, the number of sample was too small when divided by salary, work type, or assigned patient type. Further large-scale studies are needed in the future.

Recently, with growing number of elderly patients, more physical and occupational therapists are working together in hospitals. And increasing burnout syndrome with the number of therapists is emerging as a social problem. In this situation, the significance of this study lies in the fact that it examined job stress and burnout in both physical and occupational therapists at several hospitals simultaneously and identified a vulnerable group of participants notwithstanding the above limitations. 

## 5. Conclusions

Examination of the factors that influence job stress and burnout revealed that female therapists in their 20s working at small- or medium-sized hospitals were at high risk of job stress and burnout. To reduce this risk in vulnerable groups, it is necessary to understand the nature of the therapist’s work, which inevitably increases the risk of job stress and burnout; reduce workloads; improve work efficiency, and create suitable work environments. Moreover, the hospital and society should ensure on-going support of emotional health and job satisfaction for health professionals. These efforts could help to reduce the risk to vulnerable therapists and improve healthcare provision.

## Figures and Tables

**Table 1:** Job stress and burnout level according to hospital size.

Variable	Large Hospital	Small or Medium Hospital	*t* (*p*)
	*n* = 97 (30.2%)	*n* = 227 (69.8%)	
	M ± SD	M ± SD	
JCQ	1.88 ± 0.27	1.80 ± 0.26	2.651 (0.008) **
MBI			
Emotional Exhaustion	1.74 ± 0.67	2.07 ± 0.65	−4.075 (<0.001) **
Depersonalization	0.98 ± 0.58	1.38 ± 0.59	−5.636 (<0.001) **
Personal Accomplishment	3.47 ± 0.51	3.08 ± 0.06	−6.155 (<0.001) **

Note: Continuous variables were expressed as M = Mean ± SD = Standard Deviation. Parametric statistics on the hospital size and independent variable Independent *t*-test (Student’s *t*-test) analysis to job stress and exhaustion, emotional exhaustion, depersonalization, personal accomplishment in exhaustion subgroup; **—*p* < 0.01.

**Table 2:** Job stress by various variables.

Variable	Large Hospital			Small or Medium Hospital		
	*n* (%)	JCQ		*n* (%)	JCQ	
		M ± SD	*t* or F (*p*)		M ± SD	*t* or F (*p*)
			Scheffe			Scheffe
**Gender**						
Male	49 (50)	1.86 ± 0.31	0.577 (0.565)	82 (36.1)	1.89 ± 0.27	4.024 (<0.001) **
Female	49 (50)	1.90 ± 0.22		145 (63.9)	1.75 ± 0.24	
Age						
20-29	37 (37.8)	1.86 ± 0.23	−0.485 (0.629)	141 (62.1)	1.77 ± 0.24	−1.882 (0.062)
≥30	61 (62.2)	1.89 ± 0.29		86 (37.9)	1.84 ± 0.28	
Year Income						
≥50 Million Won	24 (24.5)	1.88 ± 0.27	0.715 (0.545)	2 (0.9)	2.48 ± 0.29	6.253 (<0.001) **
≥30–<50 Million Won	28 (28.6)	1.94 ± 0.32		58 (25.6)	1.83 ± 0.27	
≥20–<30 Million Won	42 (42.9)	1.85 ± 0.24		142 (62.6)	1.77 ± 0.24	
<20 Million Won	4 (4.1)	1.78±0.10		25 (11)	1.84 ± 0.26	
Education Level						
Graduate School	44 (44.9)	1.91 ± 0.30	1.004 (0.370)	46 (20.3)	1.75 ± 0.24	2.534 (0.082)
University	46 (46.9)	1.84 ± 0.20		98 (43.2)	1.81 ± 0.27	
College	8 (8.2)	1.93 ± 0.38		83 (36.6)	1.85 ± 0.25	
Marital Status						
Married	46 (46.9)	1.88 ± 0.27	0.079 (0.937)	47 (20.7)	1.83 ± 0.31	1.021 (0.309)
Single	52 (53.1)	1.88 ± 0.27		180 (79.3)	1.79 ± 0.24	
Religion						
Any religion	48 (49)	1.85 ± 0.27	−0.980 (0.330)	67 (29.5)	1.87 ± 0.25	2.781 (0.006) **
No religion	50 (51)	1.91 ± 0.27		160 (70.5)	1.77 ± 0.26	
Job Group						
Physical Therapist	64 (65.3)	1.86 ± 0.24	−1.244 (0.216)	152 (67)	1.78 ± 0.25	−0.987 (0.325)
Occupational Therapist	34 (34.7)	1.93 ± 0.31		75 (33)	1.82 ± 0.28	
Employment Type						
Regular Worker	64 (65.3)	1.90 ± 0.27	0.742 (0.460)	193 (85)	1.81 ± 0.26	1.705 (0.090)
Irregular Worker	34 (34.7)	1.85 ± 0.27		34 (15)	1.72 ± 0.21	
Work Type						
Kinesitherapy	40 (40.8)	1.84 ± 0.28	1.846 (0.127)	128 (56.4)	1.78 ± 0.25	0.549 (0.700)
Therapy for Children	19 (19.4)	2.02 ± 0.24		6 (2.6)	1.78 ± 0.20	
Electrotherapy	3 (3.1)	1.73 ± 0.20		9 (4)	1.74 ± 0.19	
Occupational Therapy	24 (24.5)	1.88 ± 0.27		73 (32.2)	1.82 ± 0.28	
Etc Therapy	12 (12.2)	1.84 ± 0.25		11 (4.8)	1.87 ± 0.23	
Employment Carrier						
≥6 yr	51 (52)	1.87 ± 0.26	0.581 (0.561)	77 (33.9)	1.82 ± 0.28	0.428 (0.652)
≥3–<6 yr	18 (18.4)	1.94 ± 0.35		53 (23.3)	1.78 ± 0.23	
<3 yr	29 (29.6)	1.85 ± 0.23		97 (42.7)	1.79 ± 0.25	
Assigned Patient Type						
CNS patient	50 (51)	1.85 ± 0.31	1.668 (0.179)	198 (87.2)	1.80 ± 0.27	0.089 (0.966)
MSK patient	10 (10.2)	1.79 ± 0.17		19 (8.4)	1.78 ± 0.19	
Children	21 (21.4)	1.89 ± 0.22		4 (1.8)	1.85 ± 0.16	
Others	17 (17.3)	2.00 ± 0.22		6 (2.6)	1.82 ± 0.16	
Experience of Turnover						
Yes	61 (62.2)	1.88 ± 0.25	−0.080 (0.936)	108 (47.6)	1.79 ± 0.27	−0.178(0.859)
No	37 (37.8)	1.88 ± 0.29		119 (52.4)	1.80 ± 0.25	
Total	97	1.88 ± 0.27		227	1.80 ± 0.26	

Note: Continuous variables are expressed as M = Mean ± SD = Standard Deviation. yr = years, CNS = Central nerve system, MSK = Musculoskeletal. Categorical variables are expressed as frequency and %. Parametric statistics on a split for the hospital size variable. Independent *t*-test (Student’s *t*-test) and ANOVA, Post-hoc Scheffe. Analysis of job stress and exhaustion according to personal and job characteristics. Personal characteristics variables include gender, age, year income, education level, marital status, and religion. Job characteristics variables include job group, employment type, work type, employment carrier, assigned patient type, and experience of turnover; **—*p* < 0.01.

**Table 3:** Burnout level by various variables in large hospitals.

Variable	Large Hospital						
	*n* (%)	Emotional Exhaustion		Depersonalization		Personal Accomplishment	
		M ± SD	*t* or F (*p*)	M ± SD	*t* or F (*p*)	M ± SD	*t* or F (*p*)
			Scheffe		Scheffe		Scheffe
**Gender**							
Male	49 (50)	1.71 ± 0.72	−0.433 (0.666)	1.05 ± 0.64	1.221 (0.225)	3.28 ± 0.54	−4.461 (<0.001) **
Female	49 (50)	1.78 ± 0.62		0.91 ± 0.51		3.58 ± 0.45	
Age							
20–29	37 (37.8)	1.95 ± 0.62	2.415 (0.018) *	1.19 ± 0.50	2.917 (0.004) **	3.56 ± 0.44	3.247 (0.001) **
≥30–39	61 (62.2)	1.62 ± 0.68		0.85 ± 0.59		3.32 ± 0.57	
Year Income							
≥50 Million Won	24 (24.5)	1.82 ± 0.65	4.491 (0.005) **	0.86 ± 0.78	2.705 (0.050)	2.38 ± 0.53	5.622 (0.001) **
≥30–<50 Million Won	28 (28.6)	1.38 ± 0.61		0.80 ± 0.43		3.33 ± 0.59	
≥20–<30 Million Won	42 (42.9)	1.90 ± 0.67		1.16 ± 0.51		3.52 ± 0.46	
<20 Million Won	4 (4.1)	2.17 ± 0.19		1.05 ± 0.34		3.57 ± 0.42	
Education Level							
Graduate School	44 (44.9)	1.63 ± 0.69	1.075 (0.345)	0.84 ± 0.65	2.738 (0.070)	3.26 ± 0.44	5.708 (0.004) **
University	46 (46.9)	1.84 ± 0.67		1.12 ± 0.51		3.49 ± 0.54	
College	8 (8.2)	1.82 ± 0.50		0.95 ± 0.37		3.56 ± 0.47	
Marital Status							
Married	46 (46.9)	1.62 ± 0.65	–1.759 (0.082)	0.81 ± 0.46	−2.801 (0.006) **	3.27 ± 0.53	−3.018 (0.003) **
Single	52 (53.1)	1.85 ± 0.68		1.13 ± 0.64		3.52 ± 0.49	
Religion							
Any religion	48 (49)	1.68 ± 0.71	−0.879 (0.382)	0.95 ± 0.68	−0.389 (0.698)	3.36 ± 0.47	−2.045 (0.042) *
No religion	50 (51)	1.80 ± 0.64		1.00 ± 0.47		3.51 ± 0.52	
Job Group							
Physical Therapist	64 (65.3)	1.78 ± 0.25	0.675 (0.501)	1.03 ± 0.65	1.111 (0.269)	3.47 ± 0.53	−0.144 (0.885)
Occupational Therapist	34 (34.7)	1.82 ± 0.28		0.89 ± 0.42		3.48 ± 0.47	
Employment Type							
Regular Worker	64 (65.3)	1.71 ± 0.66	−0.786 (0.434)	0.89 ± 0.58	−1.990 (0.049) *	3.45 ± 0.52	−1.386 (0.167)
Irregular Worker	34 (34.7)	1.82 ± 0.69		1.14 ± 0.55		3.58 ± 0.45	
Work Type							
Kinesitherapy	40 (40.8)	1.71 ± 0.78	0.418 (0.795)	1.10 ± 0.73	1.094 (0.364)	3.50 ± 0.52	1.423 (0.227)
Therapy for Children	19 (19.4)	1.89 ± 0.70		0.79 ± 0.47		3.50 ± 0.53	
Electrotherapy	3 (3.1)	2.00 ± 0.58		1.13 ± 0.23		3.22 ± 0.57	
Occupational Therapy	24 (24.5)	1.69 ± 0.55		0.95 ± 0.42		3.49 ± 0.47	
Etc Therapy	12 (12.2)	1.68 ± 0.53		0.88 ± 0.43		3.21 ± 0.54	
Employment Carrier							
≥6 yr	51 (52)	1.69 ± 0.70	0.726 (0.487)	0.87 ± 0.62	2.326 (0.103)	3.36 ± 0.59	5.027 (0.007) **
≥3–<6 yr	18 (18.4)	1.70 ± 0.69		1.01 ± 0.50		3.40 ± 0.49	
<3 yr	29 (29.6)	1.87 ± 0.60		1.15 ± 0.53		3.59 ± 0.42	
Assigned Patient Type							
CNS patient	50 (51)	1.69 ± 0.71	2.407 (0.072)	0.98 ± 0.67	1.558 (0.205)	3.50 ± 0.50	1.805 (0.147)
MSK patient	10 (10.2)	1.80 ± 0.17		1.20 ± 0.65		3.30 ± 0.54	
Children	21 (21.4) (21.4)	2.05 ± 0.62		1.05 ± 0.45		3.53 ± 0.58	
Others	17 (17.3) (17.3)	1.50 ± 0.46		0.74 ± 0.28		3.13 ± 0.42	
Experience of turnover							
Yes	61 (62.2)	1.62 ± 0.71	−2.453(0.016) *	0.92 ± 0.57	−1.381 (0.171)	3.36 ± 0.56	−2.983 (0.003) **
No	37 (37.8)	1.95 ± 0.55		1.08 ± 0.58		3.57 ± 0.43	
Total	97	1.74 ± 0.67		0.98 ± 0.58		3.47 ± 0.51	

Note: Continuous variables are expressed as M=Mean ± SD=Standard Deviation. Categorical variables are expressed as frequency and %. Use parametric statistics for a split to the hospital Size Variable was an Independent *t*-test and ANOVA, Post-hoc Scheffe. Analysis of MBI subscale (emotional exhaustion, depersonalization, personal accomplishment) according to personal and job characteristics. Personal characteristics variables include gender, age, year income, education level, marital status, and religion. Job characteristics variables include job group, employment type, work type, employment carrier, assigned patient type, and experience of turnover. CNS = Central nerve system, MSK = Musculoskeletal; *—*p* < 0.05; **—*p* < 0.01.

**Table 4:** Burnout level by various variables in small or medium hospitals.

Variable	Small or Medium Hospital						
	*n* (%)	Emotional exhaustion		Depersonalization		Personal Accomplishment	
		M ± SD	*t* or F (*p*)	M ± SD	*t* or F (*p*)	M ± SD	*t* or F (*p*)
			Scheffe		Scheffe		Scheffe
**Gender**							
Male	82 (36.1)	1.82 ± 0.70	−4.307 (0.001) **	1.21 ± 0.61	−3.331 (0.001) **	3.11 ± 0.58	−0.510 (0.611)
Female	145 (63.9)	2.21 ± 0.57		1.47 ± 0.56		3.05 ± 0.56	
Age							
20-29	141 (62.1)	2.17 ± 0.61	3.037 (0.003) **	1.43 ± 0.59	1.769 (0.078)	2.97 ± 0.54	2.496 (0.014) *
≥30-39	86 (37.9)	1.90 ± 0.67		1.29 ± 0.58		3.26 ± 0.58	
Year Income							
≥50 Million Won	2 (0.9)	0.94 ± 0.71	7.996 (<0.001) **	0.40 ± 0.28	4.467 (0.005) **	2.81 ± 0.54	3.228 (0.026) *
≥30–<50 Million Won	58 (25.6)	1.81 ± 0.71		1.23 ± 0.59		3.05 ± 0.51	
≥20–<30 Million Won	142 (62.6)	2.20 ± 0.58		1.46 ± 0.56		3.22 ± 0.59	
<20 Million Won	25 (11)	1.96 ± 0.62		1.28 ± 0.63		3.38 ± 0.27	
Education Level							
Graduate School	46 (20.3)	1.94 ± 0.71	1.705 (0.184)	1.26 ± 0.55	1.360 (0.259)	2.83 ± 0.49	9.210 (<0.001) **
University	98 (43.2)	2.05 ± 0.62		1.38 ± 0.62		3.29 ± 0.56	
College	83 (36.6)	2.16 ± 0.64		1.44 ± 0.56		3.22 ± 0.46	
Marital Status							
Married	47 (20.7)	1.80 ± 0.76	−3.209 (0.002) **	1.29 ± 0.69	−1.003 (0.320)	2.99 ± 0.55	−1.509 (0.134)
Single	180 (79.3)	2.14 ± 0.60		1.40 ± 0.56		3.16 ± 0.57	
Religion							
Any religion	67 (29.5)	2.11 ± 0.69	0.627 (0.531)	1.31 ± 0.58	−1.192 (0.234)	3.03 ± 0.55	−0.794 (0.429)
No religion	160 (70.5)	2.05 ± 0.63		1.41 ± 0.59		3.12 ± 0.58	
Job Group							
Physical Therapist	152 (67)	2.10 ± 0.65	1.031 (0.304)	1.42 ± 0.60	1.498 (0.136)	2.97 ± 0.55	−2.590 (0.011) *
Occupational Therapist	75 (33)	2.00 ± 0.64		1.29 ± 0.55		3.28 ± 0.55	
Employment Type							
Regular Worker	193 (85)	2.06 ± 0.65	−0.598 (0.550)	1.34 ± 0.57	−2.371 (0.019) *	3.03 ± 0.56	−1.211 (0.229)
Irregular Worker	34 (15)	2.13 ± 0.64		1.59 ± 0.65		3.17 ± 0.58	
Work Type							
Kinesitherapy	128 (56.4)	2.11 ± 0.63	2.157 (0.075)	1.42 ± 0.58	0.613 (0.654)	2.98 ± 0.59	1.923 (0.113)
Therapy for Children	6 (2.6)	1.80 ± 0.85		1.53 ± 0.52		3.01 ± 0.50	
Electrotherapy	9 (4)	2.48 ± 0.61		1.38 ± 0.90		3.25 ± 0.65	
Occupational Therapy	73 (32.2)	2.01 ± 0.63		1.29 ± 0.55		3.34 ± 0.53	
Etc Therapy	11 (4.8)	1.76 ± 0.75		1.38 ± 0.68		2.96 ± 0.57	
Employment Carrier							
≥6 yr	77 (33.9)	1.93 ± 0.69	3.593 (0.029) *	1.31 ± 0.61	1.321 (0.269)	2.97 ± 0.55	2.610 (0.079)
≥3–<6 yr	53 (23.3)	2.23 ± 0.64		1.48 ± 0.62		3.08 ± 0.50	
<3 yr	97 (42.7)	2.09 ± 0.60		1.37 ± 0.55		3.27 ± 0.60	
Assigned Patient Type							
CNS patient	198 (87.2)	2.07 ± 0.66	1.145 (0.332)	1.36 ± 0.57	0.411 (0.745)	3.01 ± 0.62	1.096 (0.355)
MSK patient	19 (8.4)	1.91 ± 0.45		1.45 ± 0.66		3.25 ± 0.56	
Children	4 (1.8)	2.11 ± 0.87		1.55 ± 0.66		3.22 ± 0.47	
Others	6 (2.6)	2.46 ± 0.37		1.53 ± 0.95		3.02 ± 0.52	
Experience of Turnover							
Yes	108 (47.6)	2.06 ± 0.71	−0.142 (0.887)	1.36 ± 0.62	−0.414 (0.680)	3.01 ± 0.59	−1.476 (0.143)
No	119 (52.4)	207 ± 0.59		1.39 ± 0.56		3.19 ± 0.52	
Total	227	2.07 ± 0.65		1.38 ± 0.59		3.08 ± 0.57	

Note: Continuous variables are expressed as M = Mean ± SD = Standard Deviation. Categorical variables are expressed as frequency and %. Use parametric statistics for a split to the hospital Size Variable was Independent *t*-test and ANOVA, Post-hoc Scheffe. Analysis of MBI subscale (emotional exhaustion, depersonalization, personal accomplishment) according to personal and job characteristics. Personal characteristics variables include gender, age, year income, education level, marital status, and religion. Job characteristics variables include job group, employment type, work type, employment carrier, assigned patient type, and experience of turnover. CNS = Central nerve system, MSK = Musculoskeletal; *—*p* < 0.05; **—*p* < 0.01.
